# “It is all about the fear of being discriminated [against]…the person suffering from HIV will not be accepted”: a qualitative study exploring the reasons for loss to follow-up among HIV-positive youth in Kisumu, Kenya

**DOI:** 10.1186/1471-2458-14-1154

**Published:** 2014-11-06

**Authors:** Hilary T Wolf, Bonnie L Halpern-Felsher, Elizabeth A Bukusi, Kawango E Agot, Craig R Cohen, Colette L Auerswald

**Affiliations:** Department of Pediatrics, Georgetown University Medical Center, 4200 Wisconsin Ave. NW, Washington, DC 20026 USA; Department of Pediatrics, Stanford University Palo Alto, Stanford, CA USA; Family AIDS Care and Education Services-Kenya Medical Research Institute, Nairobi, Kenya; Impact Research and Development Organization, Kisumu, Kenya; Department of OB/GYN, University of California at San Francisco, San Francisco, CA USA; UC Berkeley-UCSF Joint Medical Program, UC Berkeley School of Public Health, Berkeley, Berkeley, CA USA

**Keywords:** Adolescent, HIV/AIDS, Kenya, Lost to follow-up, Stigma

## Abstract

**Background:**

Youth represent 40% of all new HIV infections in the world, 80% of which live in sub-Saharan Africa. Youth living with HIV (YLWH) are more likely to become lost to follow-up (LTFU) from care compared to all other age groups. This study explored the reasons for LTFU among YLWH in Kenya.

**Methods:**

Data was collected from: (1) Focus group Discussions (n = 18) with community health workers who work with LTFU youth. (2) Semi-structured interviews (n = 27) with HIV + youth (15–21 years old) that had not received HIV care for at least four months. (3) Semi-structured interviews (n = 10) with educators selected from schools attended by LTFU interview participants. Transcripts were coded and analyzed employing grounded theory.

**Results:**

HIV-related stigma was the overarching factor that led to LTFU among HIV + youth. Stigma operated on multiple levels to influence LTFU, including in the home/family, at school, and at the clinic. In all three settings, participants’ fear of stigma due to disclosure of their HIV status contributed to LTFU. Likewise, in the three settings, the dependent relationships between youth and the key adult figures in their lives were also adversely impacted by stigma and resultant lack of disclosure. Thus, at all three settings stigma influenced fear of disclosure, which in turn impacted negatively on dependent relationships with adults on whom they rely (i.e. parents, teachers and clinicians) leading to LTFU.

**Conclusions:**

Interventions focusing on reduction of stigma, increasing safe disclosure of HIV status, and improved dependent relationships may improve retention in care of YLWH.

## Background

HIV is a leading cause of morbidity and mortality among youth 15–24 years of age in the developing world [1]. Youth represent 40% of all new HIV infections worldwide [2, 3], 60% of which are in sub-Saharan Africa (SSA) [3]. A western province in Kenya, Nyanza, has an HIV prevalence of 15.1%, which is three times greater than the overall HIV prevalence in Kenya of 5.6% [4]. The prevalence of HIV among adolescents 15–24 years of age in Nyanza is 8% (~200,000 people) [5]. Given the enormous burden of HIV-related disease among youth in SSA and their need for life-long treatment, early enrollment and retention in HIV care is essential [6]. If youth living with HIV (YLWH) do not receive consistent health care, they are at risk for anti-retroviral (ARV) treatment failure, opportunistic infections, increased risk of sexual HIV transmission, and ultimately premature death [7–9].

Youth are less likely than adults and children to be tested for HIV, access HIV-related care, remain in care, and achieve viral suppression [3, 10]. Prior research in Nyanza has found that 57% of the HIV-positive adolescent patients in this community were lost to follow-up (LTFU) (Otieno, unpublished manuscript). While retention in care is a global challenge affecting the developed and developing world [7], there is a paucity of research regarding the challenges HIV positive youth living in SSA face.

Research among other populations has shown that adherence to ARVs is particularly difficult due to: 1) fear of disclosure 2) HIV-related stigma, 3) poverty, 4) mental health, and 5) insufficient support networks [11–17]. There have been few studies in SSA that explore barriers to ARV adherence in YLWH. However, one study in Rwanda found that HIV-related stigma, inadvertent disclosure, poor support, lack of privacy in living situations and desire to be ‘normal’ influenced adherence to ARVs [18]. It is important to determine if these factors play a role in LTFU for YLWH in Kenya. Research with adults in SSA has found that HIV-related stigma and discrimination by healthcare providers, family members, and the community at large are major factors hindering HIV-positive patients from accessing care [17]. These factors likely play a role in LTFU for YLWH in SSA as well. However, few studies have shown how HIV-related stigma affects YLWH in SSA.

It has also been hypothesized that structural factors, such as the school environment, may play a role in the retention of care of YLWH. A study in South Africa among school-going YLWH found that many youth had not disclosed their HIV status to their teachers for fear of stigma and discrimination [19]. Targeting schools could be important for youth in SSA, as they spend the majority of their waking hours in school which they attend up to six days a week [20]. Prior research suggests that the school environment is an ideal setting to implement interventions to improve the uptake and retention of HIV care by youth [21] however, there is a paucity of literature exploring this.

Understanding the reasons for LTFU among HIV-positive youth in SSA will provide critical information to design feasible, acceptable and effective interventions for this large population of HIV-infected individuals who are less likely to access and remain in health care compared to other populations. Through the triangulation of focus groups and interviews with health care workers, HIV positive youth, and educators in SSA, we identified and explored the multi-level factors contributing to LTFU among YLWH in SSA.

## Methods

### Setting

This study took place in Kisumu, previous capital of Nyanza Province, and under the new Kenyan constitution, the headquarters for Kisumu County. Nyanza Province is one of Kenya’s poorest areas with 63% of the population living on less than $1 a day [22]. The majority of youth living in this community lack money for basic needs. Comprehensive HIV care is free of charge, but lack of transport to clinic and money for food are known barriers to obtaining care.

### Participants

We conducted a qualitative study employing focus group discussions (FGDs) with community health workers (CHW; n = 18); semi-structured interviews with YLWH (15–21 years of age) who were LTFU (defined as no contact with the clinic for four months or greater during the past year; n = 28); and semi-structured interviews with educators (including teachers, principals, or other school administrators; n = 10).

### Procedures

FGD participants were recruited using purposive sampling from four government-run HIV clinics in Kisumu. CHWs (some of which were HIV infected) were eligible if they had been working as a health worker for at least three months, and reported working with HV infected youth clients. LTFU youth participants were recruited from two clinics that served youth. A list was generated from the clinics electronic medical record of all LTFU patients in the target age range. Youth were invited to participate either by phone or in person during a home visit by a CHW on the study team. However, all interviews were conducted face-to-face. Educators were recruited from schools attended by LTFU participants. Eligible educators had to have been working at the school for at least six months. We collected a list of the schools LTFU participants had attended or were attending, and then selected an educator from 10 schools where LTFU participants were currently attending or had attended school in the past two years. The principal from each school was approached and asked to nominate an educator from their school who was knowledgeable regarding the needs of HIV infected students. The nominated educator was then asked if he or she wanted to participate in the study.

FGDs were led by trilingual Kenyan research assistants and conducted predominantly in English. Although participants were fluent in English, at times Dholuo and Kiswahilli phrases were interjected as is common in Kenya. Individual interviews were conducted by a Kenyan research assistant in English, Kiswahilli, or Dholuo based on participants’ language preference.

### Protection of human subjects

All participants provided written informed consent, or assent if they were minors, to participate in the study and were reimbursed for transportation expenses. Parental consent for youth 15–17 years of age was waived in order to protect youth who may not have disclosed their HIV status to their guardians. All youth were given a multiple-choice test to assess their understanding of the consent prior to beginning the interview. Participants had to score 75% or greater in order to be eligible to participate. During the recruitment phase, LTFU youth were approached by a trained CHW wearing street clothes, who did not identify her affiliation with the clinic until she was in a private place with the potential participant. The CHW gave the participant a choice for the location of the interview. She then explained that if the participant chose to have the interview in his/her home, the interview would be held with her alone but that other people in the home may nevertheless hear the interview. The CHW encouraged the potential participant to have the interview in a private space outside the home if possible (All but two LTFU participants elected to be interviewed outside the home). All LTFU participants were referred back to their health center of choice, regardless of whether or not they completed the interview. During the recruitment of school educators, there was no mention of the reason the school was chosen for recruitment, or of the name of the student participant. School educators were told, “We are interviewing educators in the Kisumu area”. There was no contact between the study staff and LTFU participants in the school. Consent was obtained from LTFU participants for permission to visit the last school they attended for the purpose of conducting interviews with educators. The study was approved by the Institutional Review Boards at the Kenyan Medical Research Institute and the University of California at San Francisco. Permission was also obtained from the Kenyan Ministry of Education to conduct interviews in the schools.

### Measures

Prior to the individual interviews, LTFU youth participants completed a brief questionnaire, including items eliciting information about their age, gender, marital status, last clinic visit, name of last school attended, and last grade and year enrolled in school. Demographic information was not collected from CHWs or school educators. Mode of HIV transmission is not routinely collected in the HIV clinics in Kisumu. Although LTFU participants were asked during the interviews if they knew how they became infected with HIV, many denied knowing. YLWH were asked about their current living situation. The majority of the guardians who lived with the YLWH were not the youth’s biological guardians.

The FGD guide included questions regarding: 1) barriers to follow-up for YLWH, 2) enablers to follow-up, 3) the effect of the school environment on follow-up, and 4) interventions that could improve retention. The interview guide for LTFU youth participants included questions regarding individual, family, peer, school, and environmental barriers to follow-up. The interview guide for the educators included questions regarding school-based factors that may act as barriers or enablers to retention in care.

### Analysis

Each FGD and individual interview was audiotaped, transcribed and translated into English when necessary. A structured codebook and subsequent analyses were developed, guided by grounded theory [23, 24]. We began with open coding to capture maximum detail and complexity in the data [25]. The framework for the initial codebook derived from the open coding was based on major topical headings specified in the interview guide. Using the initial codebook, two of the investigators (HW, CA) coded a randomly selected focus group and five individual interview transcripts together. The initial coding classification system was refined based on coding discrepancies and discussion of potential revisions for the coding structure [25]. Additional topics pertinent to reasons for LTFU that emerged in the focus groups and individual interviews were added to the coding classification scheme, leading to the final codebook. The codebook included definitions for the classification codes and coding guidelines with illustrative examples for each code [25]. The first author coded all of the transcripts in Atlas.ti (Atlas.ti version 7, Berlin, Germany) with a co-investigator (CA) checking random transcripts for coding consistency and code drift. Using the final coded data, reports of codes were generated for secondary coding. These were reviewed in discussions amongst the investigators (HW, CA) and findings were summarized in memos. A model of factors affecting LTFU was generated and continually revised based on memos and ongoing analysis until it was finalized. Validity of the results was checked in two community forums (separate forums were held for LTFU participants and educators) where the results of the study were reported back to the interview participants. The results were discussed and the participants expressed agreement with the interpretation of the results.

## Results

### Sample

All of the 18 CHWs who met inclusion criteria and who were approached to participate in the study agreed to participate. Of the 11 school educators who met inclusion criteria and were approached to participate, 10 agreed to participate. Of the 30 LTFU participants who met inclusion criteria and were located and approached by a CHW, three refused to participate, leaving a total of 27 completed interviews. LTFU participants were 70% (19) female. Nineteen percent (5) were 16 to 17, 37% (10) 18 to 19, and 44% (12) 20 to 21 years of age. Fifty-nine percent (16) self identified as single, 26% (7) as married, and 15% (4) as separated. Twenty-six percent (7) of youth were attending school at the time of the interview.

### Grounded theory model

**Figure 1:**
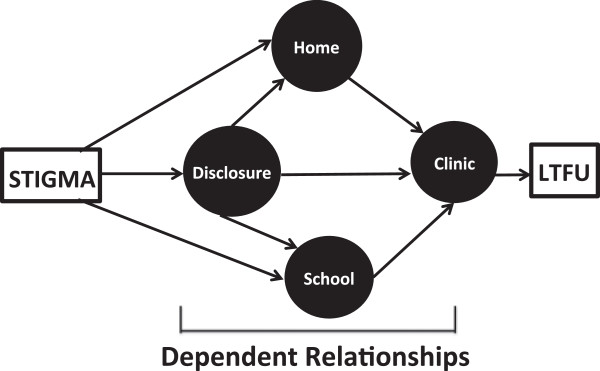
**Grounded theory model.**

Our model is illustrated in Figure 1. In brief, HIV-related stigma was the overarching factor that led to LTFU among YLWH. HIV-related stigma operated on multiple levels to influence LTFU at home, school, and in clinic settings. In all three settings, participants’ fear of the stigma that could result from disclosure of their status negatively impacted on their dependent or fiduciary relationships with adult caretakers (including parents, teachers, and clinicians) leading to LTFU. We define dependent or fiduciary relationships as supportive caring relationships between youth and adults charged with the youth’s growth and wellbeing [26].

Participants described multiple types of HIV-related stigma in all three settings. These included: internalized stigma, or instances when youth stigmatize themselves because of their HIV status; perceived stigma, whereby youth feel that someone is stigmatizing them; fear of enacted stigma, which is expressed when youth act to avoid being discriminated against because of their HIV status; and enacted stigma, defined as experiencing discrimination based on one’s HIV status.

### The home environment and LTFU

HIV-related stigma, fear of disclosure and compromised dependent relationships with relatives or guardians contributed to LTFU. In a large part due to the HIV-related mortality, but also due to all-cause mortality, relatives other than biological parents were raising most study participants. When a parent died, it was expected that a relative would parent the orphaned child. However, as stated by an 18 year-old female participant, “It is always rare to get a person who is ready to help you out after the death of your parents”. These circumstances often resulted in poor dependent relationships, stigma directed toward YLWH by family members and fear of disclosure to family members.

#### Family HIV-related stigma

The majority of YLWH experienced HIV-related stigma from family members. This was often enacted stigma based on misinformation about HIV that led to discrimination and poor mental health outcomes. A CHW discussed family-based stigma regarding beliefs about HIV transmission. *“In a home set up, you will find that if it is a mother taking care of an orphan…the utensils they are using in the house will be separated…*[*Stigma*] *comes in the things that people use”.*

Many participants discussed families’ beliefs that an HIV diagnosis was a death sentence, with implications regarding support, particularly educational support, for infected youth. A 20 year-old (y.o.) LTFU female said, “*I told* [*my uncle*] *that I wanted to go back to school but he said that I was dead long time ago. There is no need of taking a sick person to the school”.*

HIV-positive youth recounted the negative impact perceived family HIV-related stigma had on their mental health. One 18 y.o. LTFU female stated, *“You will become lonely because* [*your family*] *will reject you”.* Another 20 y.o. LTFU female said, *“Some of the fathers*…*will mishandle you*…*This will drive a patient into suicide”.*

#### Disclosure to family

Fears of having family members discover one’s HIV status influenced participants’ clinic follow-up and their medication adherence for the majority of YLWH who had been diagnosed with HIV in the past 3 years. One19 y.o LTFU female responded, “*At times*, *you are unable to inform the parents about* [*your HIV status*]. *Even if you are given the drugs*, *you cannot get the opportunity to use them*”. One 18 y.o. LTFU female believed that family members spy on youth to discover their status,

*Your parents will demand to know where you are headed to. You will tell them that you are going to the hospital. They will check the content of your bag. They will be lucky to get the drugs.*

Some family members were aware of their youth’s HIV status, especially the family members of participants who had been diagnosed with HIV at a younger age, suggesting vertical transmission. However, these family members did not always keep the information confidential, as illustrated by this quote from a 21 y.o. LTFU female,

*You might be staying with your stepmother who has her own children in the same house. These children might decide to disrespect you*. ‘*Leave alone that guy. He is infected with HIV*/*AIDS,’ they will say*.

#### Family-related support

Participants also reported positive familial support they received to stay in care, including monetary support, emotional support, and support regarding keeping their status confidential. Some participants, mostly older youth, focused primarily on monetary support. “*My sister used to send me* [*money*] *so that I can honor my clinic appointment. We can use the money to pay for my transport*” (21 y.o. LTFU female). In addition to monetary support, younger participants focused on the emotional support they received including love, care, and encouragement.

Emotional support appeared to play a role in improving participants’ mental health. Multiple participants felt supported by their family if they helped them remember their clinic appointments and drugs, and discussed their infection with them openly. For example, one 17 y.o. LTFU male said, “*Even if I forget about the drugs*, [*my guardians*] *remind me to take them. I feel more encouraged when someone reminds me about the drug*”. Older participants discussed familial emotional support less frequently than younger participants.

Conversely, some participants discussed their lack of familial emotional support. One 18 y.o. LTFU male stated that his emotional support from his sister, also his guardian, was so poor that he stopped adhering to medication and seeking care. *“My sister*, *she usually treats me rough. Sometimes I get somewhere to sit and think*, *is this really my sister*?*”* When this participant was asked why he was not adhering to his medications and following up in care, he answered, *“It is only my sister”.*

##### Support regarding confidentiality

Participants stressed the importance of secrecy and how families can be supportive by keeping a youth’s status confidential. One 17 y.o male participant said, “*I don*’*t go through difficulties because my guardians were the first people to know about my status and they didn*’*t tell anyone*”.

#### Dependent relationships at home

Although some youth had supportive families, many LTFU participants reported having guardians who did not fulfill their caretaker role for their dependent youth, did not treat youth’s medical care as a priority and who therefore did not assist them in accessing and staying in care as shown in the quote below.

16 y.o. male: *At home, you will find that people are aware of [my HIV status] but instead of telling you that you should go to the clinic, they also forget it”.*

Interviewer: *Does [your uncle] show any concern to help you with this?”*

16 y.o. male: *No. I just feel that he is being forced…In terms of education, they support me. However, they just [disclosed my HIV status to me] and let me [take care of myself]”.*

This student also had difficulty with his guardians helping him obtain permission at school to attend clinic. The boy said, “*[My guardians] have no time to come to school. No one even came the last time that they were called”.* Educators also commented on the lack of proper dependent relationships at home. One teacher said, “*I think most parents are not aware of how to take care of the children. They don’t know what the child requires. I think the parents are not taking their part effectively”*.

For youth who are minors living with their parents or guardians, for whose care Kenyan law requires guardian permission, the lack of support from their guardians directly affected their ability to follow-up in clinic. Without guardian support, these youth encountered many barriers to attending clinic, suffered from lack of advocacy during clinic visits, were unable to adhere to medications, and lacked guidance in negotiating excusal and the dispensations needed in the school setting to attend appointments.

### School environment and LTFU

Like at home, HIV-related stigma, disclosure and compromised dependent relationships with adults at school, contributed to LTFU of HIV-positive participants. The Kenyan school environment structure greatly influenced the ability of HIV-positive students to stay engaged in medical care. Specifically, students in Kenya attend school for very long hours with some students attending school on the weekend. Students in day school often commute long distances. Boarding school students generally attend schools far from home and rarely return home during the school session. The ability of these students to remain in care was particularly dependent on their school environment. The majority of the students attended government schools, which with their strict and rigid policies, also contributed to LTFU.

#### School HIV-related stigma

##### Educator HIV-related stigma

School stigma, including educator-related and peer-related stigma was widespread. Educator-related stigma included enacted stigma, fear of enacted stigma, stigmatizing beliefs, stigmatizing lessons in the curriculum and perceived stigma. An example of educator-enacted stigma was name-calling of HIV-positive students. A 20 y.o. LTFU female said, “*The teacher will look down upon the child instead of helping him. They can call such students prostitutes*”. Another example of enacted stigma was exclusion from school, as shared in this quote by a 20 y.o. LTFU female who had not been diagnosed with HIV until after finishing school, “*Teachers used to send them away from school claiming that they might infect the others*”. In their interviews, educators expressed stigmatizing beliefs regarding HIV-positive students. One teacher stated, “*The first thing these students will know is that their future is gone. It has been destroyed completely”.* This teacher also spoke about how he believed HIV positive youth would want to spread the virus to HIV negative peers. Many of the lessons taught by teachers regarding HIV perpetuated inaccurate and stigmatizing beliefs. One teacher said,

*The solutions are obvious they have to abstain and nothing else. There is no need of telling them to use condoms. We are also telling them the dangers [of HIV]. You will die. That is one. You will suffer in this world because of that disease.*

School educators were asked how an adolescent contracts HIV. The majority of teachers put particular emphasis on sex. When educators were probed regarding other modes of transmission of HIV, many were unaware that an infant could be born with HIV and survive to adolescence. Some educators held inaccurate beliefs about youth’s infectivity. The 2011 7th grade science textbook states that HIV can be passed by saliva. One educator said *“[A student] could also get HIV after sharing the same cup*”.

Some well-meaning educators feared enacted stigma leading to negative repercussions for them if they assisted a potentially infected student. Referring to his hesitancy to reach out to an ill-appearing HIV orphan, one teacher stated, “*You cannot come out and say that I have seen this [infection] in you. You see it and leave it at that. [If I do something], then I will be the talk of everybody*”.

##### Peer HIV stigma

According to participants, peers stigmatized HIV-positive students in different ways. Like educators, peers verbally stigmatized HIV-positive students in school. One teacher said, “*Once they know that you are positive, they will give you that name…Instead of calling you by your name, they will call you ‘AIDS’”.* In some instances, YLWH participants expressed their own stigmatizing comments about other HIV-positive students. In addition peers sometimes socially isolated HIV-positive students. A teacher said, *“If at all they [disclose their HIV status], other students may start isolating them and running away from them. They think the student can infect them”.*

Some educators attempted to combat enacted peer stigma. A 19 y.o. LTFU female participant recounted,

*The teachers used to say that in a whole class, there might be one student who is HIV positive. You cannot look down upon him. He is also human like you. He is just the same as those who aren’t infected.*

##### Outcomes of school-related HIV stigma

Being the subject of stigma led to negative outcomes for youth, including lack of disclosure, decisions to drop out of school, and depression. As stated by one CHW,

*In schools, many children don’t understand HIV well. People will fear you. Once everyone fears you, you will have no option but to die. You will think of eliminating yourself.*

#### School-related disclosure

The vast majority of HIV-positive students did not disclose their status at school due to fear of HIV-related stigma and of inadvertent disclosure to others. In most cases, this lack of disclosure directly affected their retention in care.

##### Disclosure to teachers and school administrators

The majority of youth chose not to disclose their HIV status to educators if they were in good health. One teacher said, *“[Students] hate disclosing. They don’t even [disclose] to their parents unless they are totally sick and are in bed. [Disclosure] can never happen when they are still healthy”.* In general LTFU participants reported that it is difficult to interact with the teachers at school, as described by this 16 y.o. male LTFU participant,

*I am not used to the teachers. I solve my problem alone…I also cannot disclose my status to them. It therefore means that the school doesn’t create an easy environment for [HIV-positive] students.*

Students’ guardians also avoided disclosing a student’s HIV status to schools, which made it difficult for YLWH to leave school to attend clinic appointments. None of the schools that we visited reported that guardians had disclosed their children’s HIV status on the health forms required for enrollment. Furthermore, although the forms ask about “illnesses”, none that we reviewed specifically asked about HIV.

##### Forced/involuntary disclosure at school

Forced or involuntary disclosure was reported in several situations. Participants reported that forced disclosure often occurred in the event of illness. An 18 y.o. LTFU male stated, “*I was developing skin rashes…my body was like in a fungal or bacteria. That is why my sister had to tell the head teacher that I am [HIV-positive]”.* Participants also reported that teachers disclosed their status against their will. At times this was done for the purpose of attempting to help a student. However in other circumstances it was harmful. Both types of breaches had the potential for further stigmatization.

Fear of involuntary disclosure, and resultant lack of disclosure directly affected poor adherence with medication in school. Furthermore, medications often needed to be taken during the students’ long school day. As described by this 17 y.o. LTFU student,

*Most of the time I spend in school and I will not like to carry my drugs to school…because maybe I can carry them to school, place them well in my bag and accidentally someone comes into my bag and opens it, finding them there. [The drugs were] making me to leave school earlier…it was bringing problems to the teachers because I didn’t tell them anything to do with my drugs.*

However, some participants reported finding teachers, guidance counselors, and even cooks at school whom they trusted and who were skilled at providing advice. These were often people not appointed by the school to serve this role but who fell into it naturally and so were sought out by students.

##### School policies contributing to issues related to disclosure

School policies regarding absenteeism, excusal from school, and missing exams were structural barriers that prevented retention in care. The overall consensus from both students and teachers was that absenteeism was inexcusable. A 16 y.o. LTFU male said, *“The first school rule states that no class lesson should be missed. If you fear disclosing, you will just remain in school”.* Educators were aware of this problem,

*A child can go to collect drugs but will not say that ‘I went for the drugs for HIV/AIDS.’ As a teacher, you will insist, ‘Why are you absent?’ This will encourage the child to avoid going for the drugs because absenteeism is not allowed* (Schoolteacher).

Most of the schools had similar procedures for obtaining permission to be excused from school. Permission was required from the principal, deputy, class teacher, and/or teacher on duty (who rotates weekly). Problems arose with this process, as one 18 y.o. LTFU male student said, “*It depends with the kind of teacher because maybe the principal or the deputy knows that you are HIV and the teacher on duty doesn’t know that”.* Some teachers granted permission for a student to leave school depending on the perceived validity of a youth’s request, particularly problematic for healthy HIV-positive students who have not disclosed their status. As an 18 y.o. LTFU female student said, “*Some teachers will want to know why you are going to the hospital yet you don’t look sick”.*

Some participants found ways to leave school for clinic without disclosing their HIV status. One boy discussed being able to go to clinic most times, but other times when exams were announced at the last minute he had to miss his clinic appointment. Even if a student was permitted to leave school, he/she may not be able to make up a missed exam. One teacher said: “*When a child has gone to the hospital, he or she might come back and find we have completed the exams. She has to pick from there…we have finished”.*

Additional issues arose related to involuntary disclosure for students attending boarding school.

One school had a policy of inspecting students’ possessions, putting them at risk for involuntary disclosure should clinic cards and medications be discovered. One participant who was a boarding master reported discovering one student’s status because he found ARVs in his bag.

#### School support

Many HIV-positive students and educators felt that in the absence of disclosure, it was not possible for a school to support HIV-positive students. A 17 y.o male student said, *“They can’t give me support when they don’t know I am a victim*”. This belief reflected students’ focus on receiving individual support from the school, such as being allowed to go to the clinic without reprisals, being excused from labor-intensive activities, or receiving nutritional supplementation.

Students focused less on the school providing an environment friendly to HIV-positive youth overall, regardless of disclosure.

Some teachers reported having taken it upon themselves to provide emotional support and counseling to individual students, as per this quote from a teacher, “*We will also try as much as possible to talk to him regularly and to make him feel comfortable that he is just like other normal students”.* However, teachers had not been provided the training for such a role.

#### Dependent relationships in school

There were instances where educators had stepped in to provide the support that students’ guardians did not provide. A 16 y.o. LTFU female described how she had been so sick that a teacher took her to clinic. When she tested positive for HIV, the teacher assisted her in attending clinic appointments, medication adherence, and with nutrition. The student was afraid to disclose her status to her guardians. When she finally did, they did not support her access to care, leaving the student to depend of her relationship with her teacher to help her access care.

Sometimes educators did not fulfill their responsibility. A boy attending boarding school was being watched over by the principal because his illness was progressing. The principal was entrusted with the boy’s medications to ensure they would be taken properly. Unfortunately problems arose from this arrangement because sometimes when the student went to take his drugs at the allotted time he would find the office locked. Additionally, unbeknownst to him, all of the teachers found out his status, eventually requiting him to leave the school.

### Clinic environment and LTFU

In addition to family and school factors, clinic-related factors contributed to LTFU, particularly clinic culture, fear of involuntary disclosure, and the failure of dependent relationships in clinic.

Patient-provider relationships in Kenya are often one-sided, with patients listening to what they are told without interjecting their preferences, including preferences regarding appointment days and times. This was typical of many clinic-related scenarios discussed by youth. Even if participants were not able to show up for their designated appointment because of a prior engagement they would not volunteer this fact. The LTFU participants feared how they would be treated for missing their previous clinic appointment, as related by this 18 y.o. LTFU female,

*It can be bad and it is also embarrassing if everyone says that you are a defaulter. You will be embarrassed because you are the center of attraction. They will say that you are visiting the clinic claiming to be sick but the real problem is that you are a defaulter. The workers at the clinic won’t treat you well. They will not assist you at this time.*

When a participant was asked about being treated badly for missing his appointment, he responded, “*As a patient, it is your fault and so you just have to bear with it” (*16 y.o. LTFU male).

#### Clinic-related disclosure

Multiple issues related to disclosure arose in the clinic setting, including fear of direct disclosure by clinic staff and fear of disclosure by association with the clinic. One 16 y.o. LTFU male said, *“I feared that the doctors were going to disclose my status to other people. I therefore decided not to come”.* One participant spoke about her fear of alleged corruption in the clinic associated with keeping ones’ status confidential. “*I am told that you have to pay some money for it to be confidential. I haven’t asked how much it will take”* (18 y.o. LTFU female). Participants recalled situations where healthcare workers were not sensitive to their confidentiality at clinics: “*At times they just shout saying, ‘Those who are here to pick the drugs should sit on that side’”* (21 y.o. LTFU female). However, one 20 y.o. LTFU female expressed trust of health care workers, “*I am sure of the client-doctor confidentiality”.* Due to fear confidentiality many participants said they preferred to be seen by healthcare professionals who did not know them.

Another frequently cited reason for not attending clinic arose from the fear of being seen and recognized in clinic, and thereby having one’s status revealed. A 17 y.o. LTFU male stated, “*As soon as I got [to clinic], I saw many of our school mates…I thought that they knew I was going to take drugs there… Since then I haven’t gone to the clinic”.* Multiple participants chose clinics far from home in an attempt to avoid being recognized, however this created other obstacles. “*You can fear going to a nearby clinic…I will have to come [to this clinic] yet I might not have [bus] fare at times”,* reported a 19 y.o. LTFU female.

#### Clinic-related support

The majority of participants cited unsupportive clinic staff and/or providers as factors contributing to their LTFU from clinic.

*I think some of the counselors there just start to shout, instead of just asking and then I give them a good reason [for missing clinic]… If they don’t relate to me good enough, I don’t think there is any reason for me to take the drugs* (17 y.o. LTFU male).

Other participants spoke highly of the care they received at their clinic and felt very supported by the clinicians, motivating them to return.

*They really make it easier. It was like my home. Maybe I wasn’t getting that attention from home. I used to come to this place and felt loved. It is like there was a chance for me to live despite my status* (20 y.o. LTFU female).

#### Dependent relationships in the clinic setting

Lack of communication between the clinic, home, and school resulted from adults’ neglect of their obligations towards youth who are dependent on them for their care. Some youth, such as this 16 y.o. LTFU male, had ideas regarding ways this problem could be approached, “*This [clinic] can go and talk to the principal. She will be reminding me to come to the clinic or she can let me go in case I approach her”.*

Participants described conflicting messages regarding their dependent relationships. As per one 17 y.o. LTFU male,

*The last time I went to the clinic…my aunt was not there and she was the one responsible for me. So, those counselors told me that the adherence session could not be done when she is not there. I was asking them is she the one who needs the drug or me. I think that time we didn’t understand each other*.

Although clinicians expected adolescents to come to clinic with their guardians and expected guardians to support youth compliance, clinicians simultaneously held youth responsible for not showing up to an appointment to the same degree they would an adult, therefore not taking into account their developmental stage and dependent role. The 17 y.o. LTFU male referred to above, added,

“*I was late maybe by one week because of exams. When I went there, they asked me, ‘Why didn’t you come?’ I told them I had exams, which I could not miss. They said even if you have an exam, you should have told us. We didn’t get on well because I told them I don’t think that’s my responsibility… I told them that I think they should call and ask why I didn’t come”.*

This participant asserted that he wanted to receive adherence counseling independently of his guardian but that nevertheless didn’t think it was his responsibility to alert the clinic that he would not be attending, suggesting that his expectations of the clinicians were also not consistent.

## Discussion

We qualitatively evaluated the reasons that YLWH aged 15–21 years are LTFU in Kisumu, Kenya. We found that LTFU was ultimately linked to fear of HIV-related stigma, which in turn inhibited disclosure and negatively impacted dependent relationships in the home, school and clinic. Adult figures in all settings often did not fulfill their responsibilities to the youth who were dependent upon them, leading to LTFU. In addition, although some YLWH wanted to be responsible for their own care, they were often unable to follow through with appointment and medication adherence. All of these themes emerged in the home, school, and clinic; suggesting that in order to address LTFU in YLWH in Kisumu interventions need to be inclusive of all three settings.

HIV-related stigma clearly emerged from this study as a major factor contributing to LTFU. Prior research has shown that the factors that contribute to HIV-related stigma among adults in SSA include fear of transmission, due to incorrect beliefs regarding the mode in which HIV is transmitted, and fear that an individual will die. Both of these stigmatizing beliefs were pertinent to youth in this study [27]. In addition youth in this study described how HIV stigma influenced depression and suicidal ideation, lack of adherence to medication and LTFU. Prior quantitative studies have shown that HIV-related stigma has a damaging effect on health outcomes among people living with HIV (PLWH), including depression [28–31]. HIV stigma has also been shown to negatively affect adherence and retention in care in a multitude of prior studies among youth in Western countries and adults in SSA [18, 27, 31–34]. This is one of the first studies to document the importance and often paralyzing effect of HIV-related stigma on retention in care for YLWH in SSA.

The school setting had a particularly profound effect on LTFU. School policies and stigmatizing beliefs among educators and students greatly impacted YLWH, making it difficult for them to attend school and to stay in care. Furthermore this study suggests that the school influenced how all students were socialized into maintaining stigmatizing beliefs about HIV due to a clear lack of scientific information in the school curriculum demythologizing HIV. This study confirms published findings that educators lack proper training regarding HIV and at times breached students’ confidentiality regarding their HIV status [18, 20, 35, 36]. However, this study takes this concept a step further by suggesting that the school environment itself in Kisumu influenced LTFU among YLWH. Despite the fact that the majority of participants in this study believed support was impossible without disclosure, support can be provided to YLWH by creating a welcoming environment free of stigma [35, 36]. If the environment is not supportive, youth and families cannot be expected to be forthcoming in their disclosures. Indeed, parents’ lack of reporting of youth’s status could be seen as protective within the current environment.

Clinic appointment adherence depends on disclosure. However, we found that the reasons for lack of disclosure were often related to fear of stigma at home and at school. The majority of participants in this study chose not to disclose their HIV status to their families or their teachers. Prior research has shown that PLWHA who voluntarily disclose their HIV status have better physical health outcomes [37, 38]. However, research has also found that uncontrolled disclosure of serostatus among adults and adolescents in SSA and western countries was associated with non-adherence to HIV medications [18, 37, 38]. This reveals the importance of encouraging controlled disclosure by creating safe and confidential environments that ensure retention in care and ultimately improve health outcomes. This study showed that lack of privacy to store and take medications at home and at school was a barrier to medication adherence, which is consistent with findings from a Rwandan study amongst YLWH [18].

HIV-related stigma, disclosure, and poor dependent relationships in the home, school and clinic accounted for the LTFU of the majority of our HIV infected youth participants. Greater social support has been shown to improve adolescents’ motivational readiness to adhere to medications in western countries [18, 28, 29, 32, 39]. In addition, HIV-infected youth attending clinics in the U.S. display a high level of trust in their current pediatric providers, therefore making transition to adult providers difficult [32]. In contrast, participants in our study cited poor relationships with healthcare providers. Participants said they wanted to see healthcare providers who did not know them because they believed this would keep their status anonymous. This exemplifies the lack of trusting relationships between youth and providers, and the degree of perceived HIV-related stigma that exists in this population. Outside of a few youth-designated clinics in Kenya, health workers receive little education regarding youth-specific issues. In many clinics youth under 15 y.o. are treated as children while youth 15 and over are treated as adults. This system does not take into account the unique challenges adolescents face and their need for adult support into late adolescence to stay in care. This predicament was illustrated in the results of this study, showing the confusion among youth, guardians and clinicians regarding who should be responsible to ensure that a YLWH returns for care.

Certain limitations are inherent in qualitative research. In particular, caution is warranted in generalizing our findings given our relatively small sample of participants. In addition, selection bias may have impacted the study results. Specifically, we were unable to locate or recruit many LTFU youth because they had moved away or we had incomplete or incorrect locator information. In addition participating educators were nominated by school principals, possibly creating a bias towards the selection of educators who were more sensitive to issues related to YLWH. These limitations notwithstanding, our qualitative findings highlight important reasons for LTFU and possible interventions to improve retention.

## Conclusions

This study has implications for the role of educators, families and healthcare providers in addressing the needs of YLWH, including the reduction of HIV stigma, provision of safe confidential environments, and recognition of youth’s developmental needs. Our results suggest that HIV stigma interventions should be implemented in clinics and schools and should be provided to families and communities in order to decrease barriers to youth’s access and retention in HIV-care. The reduction of HIV stigma may also improve safe disclosure, thereby also enabling youth to seek HIV care. However, disclosure should not be a requirement for the provision of a safe environment for YLWH to attend clinics and schools. Important adults in youths’ lives, including guardians, educators, and clinicians need to be trained regarding their shared responsibility for the well being of YLWH. Future studies should pilot interventions focusing on HIV stigma reduction and education and the training of guardians, school educators, and healthcare providers regarding the unique needs of YLWH, and collaboration of adults to improve youth’s access to care. The goal of these interventions should be to reduce HIV stigma, improve support, improve health care services for YLWH, provide a greater level of confidentiality for YLWH, decrease youth’s morbidity and mortality and optimize their wellbeing.

## Authors’ information

HTW was an Adolescent Medicine, Reproductive Infectious Disease, and Global Health fellow at University California, San Francisco (UCSF) when this study was conducted. HTW conducted this research while living in Kisumu Kenya over a period of 18 months. After finishing her fellowship, HTW joined the faculty at Georgetown University as an Assistant Professor in Adolescent Medicine. HTW had strong mentorship and research support from senior co-investigators in Kenya (EAB, KEA) and in the US (BLH, CRC, CLA).

## Abbreviations

ARV: Anti-retroviral
CHW: Community health worker
FGD: Focus group discussion
LTFU: Lost to follow-up
PLWH: People living with HIV
SSA: Sub-Saharan Africa
YLWH: Youth living with HIV
y.o: Years old.
